# Tetrabenazine as anti-chorea therapy in Huntington Disease: an open-label continuation study. Huntington Study Group/TETRA-HD Investigators

**DOI:** 10.1186/1471-2377-9-62

**Published:** 2009-12-18

**Authors:** Samuel Frank

**Affiliations:** 1 72 East Concord St., C329 Boston, MA 02118, USA

## Abstract

**Background:**

Tetrabenazine (TBZ) selectively depletes central monoamines by reversibly binding to the type-2 vesicular monoamine transporter. A previous double blind study in Huntington disease (HD) demonstrated that TBZ effectively suppressed chorea, with a favorable short-term safety profile (*Neurology *2006;66:366-372). The objective of this study was to assess the long-term safety and effectiveness of TBZ for chorea in HD.

**Methods:**

Subjects who completed the 13-week, double blind protocol were invited to participate in this open label extension study for up to 80 weeks. Subjects were titrated to the best individual dose or a maximum of 200 mg/day. Chorea was assessed using the Total Maximal Chorea (TMC) score from the Unified Huntington Disease Rating Scale.

**Results:**

Of the 75 participants, 45 subjects completed 80 weeks. Three participants terminated due to adverse events (AEs) including depression, delusions with associated previous suicidal behavior, and vocal tics. One subject died due to breast cancer. The other 26 subjects chose not to continue on with each ensuing extension for various reasons. When mild and unrelated AEs were excluded, the most commonly reported AEs (number of subjects) were sedation/somnolence (18), depressed mood (17), anxiety (13), insomnia (10), and akathisia (9). Parkinsonism and dysphagia scores were significantly increased at week 80 compared to baseline. At week 80, chorea had significantly improved from baseline with a mean reduction in the TMC score of 4.6 (SD 5.5) units. The mean dosage at week 80 was 63.4 mg (range 12.5-175 mg).

**Conclusions:**

TBZ effectively suppresses HD-related chorea for up to 80 weeks. Patients treated chronically with TBZ should be monitored for parkinsonism, dysphagia and other side effects including sleep disturbance, depression, anxiety, and akathisia.

**Trial Registration:**

Clinicaltrials.gov registration number (initial study): NCT00219804

## Background

Huntington disease (HD) is a hereditary, progressive neurodegenerative disease clinically characterized by a triad of chorea, cognitive symptoms and behavioral changes. Although there is no established treatment to delay the onset or forestall the progression of HD, symptomatic treatment of chorea may be beneficial in some individuals as it may have a favorable impact on motor function, quality of life and safety [1,2].

Many agents and surgical procedures have been evaluated in HD for their anti-choreic efficacy including dopamine depleting agents, dopamine antagonists, benzodiazepines, glutamate antagonists, acetylcholinesterase inhibitors, dopamine agonists, anti-seizure medications, cannabinoids, lithium, deep brain stimulation, and fetal cell transplantation [3-5]. A preponderance of the mostly uncontrolled studies to date supports the use of TBZ in patients with a variety of hyperkinetic movement disorders, including HD [6-8]. The efficacy of TBZ as an anti-choreic drug was convincingly demonstrated in a double-blind, placebo-controlled trial, which demonstrated clear short-term symptomatic relief of chorea [9]. There is paucity of data on the long term use of TBZ, but a few studies have provided evidence of its long-term efficacy and tolerability [7,10].

TBZ is a reversible dopamine depleting agent that is highly selective for the central vesicular monoamine transporter type 2 (VMAT2) [11]. TBZ depletes dopamine more selectively over norepinephrine and serotonin by inhibiting transport into presynaptic vesicles [12,13]. The highest binding density for TBZ is in the caudate nucleus, putamen and nucleus accumbens, areas known to bear the brunt of pathology in HD [14,15]. VMAT2 binding and monoamine depletion by TBZ is reversible, lasts hours, and is not modified by chronic treatment [16,17].

Far too little evidence is available to guide long term symptomatic treatment in HD. Double-blind and long-term studies assessing various treatment strategies in HD are urgently needed [18]. The objective of this open-label extension study was to assess the long-term safety and efficacy of TBZ in the treatment of chorea in HD.

## Methods

### Participants

Patients with HD who were ambulatory, had a Total Functional Capacity (TFC) score of greater than 5 and a Total Maximal Chorea (TMC) score of greater than 9 from the Unified Huntington Disease Rating Scale (UHDRS), were initially enrolled in the thirteen-week, double-blind, placebo-controlled study [9,19]. Subjects were excluded if they were taking concurrent dopamine depleting drugs, dopamine D_2 _receptor blockers, selective and non-selective monoamine-oxidase inhibitors, amantadine, levodopa, or dopamine agonists. Participants were permitted to be on antidepressants, antianxiety agents and other psychotropic medications at stable doses. Clinically, subjects could not have disabling dysarthria, dysphagia or depression present at screening or have an unstable or serious medical or psychiatric illness, untreated depression or lack of a caregiver.

**Figure 1:**
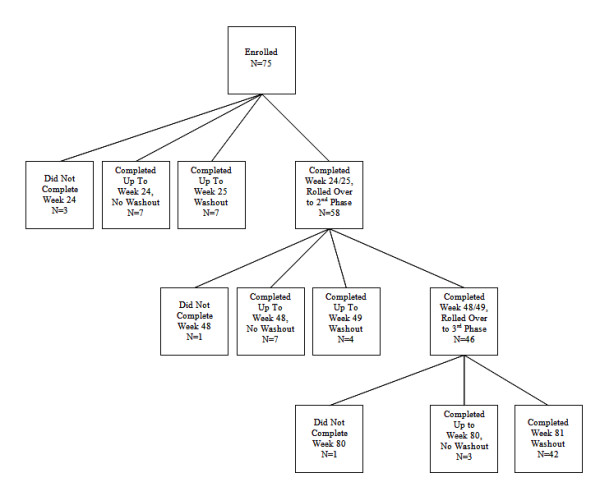
The flow of subjects (including number of subjects) through the trial from enrollment and into each of the extension phases

The subjects completed the double-blind study within eight weeks of enrollment but were subsequently excluded if they had suffered from a serious adverse event (AE) judged to be possibly or probably related to study drug. There were three possible lengths of enrollment in the open-label study (Figure 1). The initial protocol consisted of a total of 24 weeks, with 12 weeks of titration and 12 weeks of maintenance. Subjects had the option of enrolling in an extension of the 24 week study to a total of 48 weeks. At 48 weeks, they had the option of enrolling for total study duration of 80 weeks. There was a one week washout following each study phase. Since all participants had been off TBZ for at least one week following washout from the double-blind study, baseline for this study was considered to be the day of enrollment in the open-label study.

Written informed consent was obtained from all study participants and accompanying caregivers. In compliance with the Declaration of Helsinki, this study was approved by the Research Subjects Review Board at the University of Rochester as the coordinating site and by the ethics review boards at all individual sites that enrolled subjects.

#### TBZ Dosing

TBZ was titrated over a maximum of 12 weeks every 3-7 days to the best individual dose (maximum of 200 mg/day). All subjects started at 12.5 mg per day and were titrated upward at the end of each week by 12.5 mg increments to doses equal to or lower than 125 mg/day, and then by 25 mg increments for doses higher than 125 mg/day. If at any time during the titration phase, moderate to severe, possibly or probably drug-related AEs occurred, the dose of TBZ was decreased to the patient's previous well-tolerated dose. Study drug titration (up or down) was permitted only during the first 11 weeks of the study.

### Assessments

Participants were examined at the end of weeks 2, 6, 12, 24 and then every 12 weeks; and had a safety follow-up visit one week after the end of treatment. Characteristics of participants and non-participants were compared using chi-square tests and Kruskal-Wallis tests, as appropriate.

### Analysis of Efficacy Endpoints

The primary efficacy endpoint was the TMC score from the UHDRS at week 80 compared with the baseline TMC score. TMC score at week 80 was also compared to week 81 (after washout) to determine the degree of re-emergent chorea. To determine if TBZ may have worsened overall underlying chorea over time, TMC at week 81 was compared with baseline. T-tests and analysis of covariance (ANCOVA), adjusted for site and baseline value, were used to determine significance. Secondary endpoints included the Clinical Global Impression scale and the individual sections of the UHDRS.

### Analysis of Safety and Tolerability Endpoints

Tolerability was assessed using adverse events, UHDRS parkinsonism score (total of the following UHDRS motor items: finger taps, pronation/supination, rigidity, bradykinesia, gait, tandem gait and retropulsion pull test), Barnes Akathisia Scale (BAS) [20], Unified Parkinson's Disease Rating Scale (UPDRS) dysphagia and dysarthria scores [21], and the 17-item Hamilton Depression Measure (HAM) [22]. Changes from baseline were assessed using t-tests.

Treatment emergent AEs were designated to be those AEs that emerged after the start of the open-label study, excluding those for which subjects had a prior history and those that had been present during the double-blind study, including those without resolution. Those AEs that carried over from the double-blind portion of the study were not considered new events as they have been previously reported [9]. The time period examined for all AEs was baseline to last day on study medication, prior to initiation of washout.

The total number (%) of subjects experiencing at least one AE was calculated separately for the titration and maintenance periods. McNemar's test was used to compare the number of subjects experiencing at least one AE during the titration phase with the number experiencing at least one AE during the maintenance phase. AEs starting in the period from the date of baseline visit to week 12 were designated as having occurred during the titration period. AEs starting in the period from the first day of week 12 to week 24 were designated as having occurred during the maintenance period. AEs designated as mild and/or not related to study medication were excluded, and three subjects with fewer than 13.5 weeks in this study period were omitted from this analysis.

To determine if 12 additional weeks of exposure to study medication impacted chorea or HD, efficacy and safety measures were compared between subjects originally assigned to placebo and TBZ in the double-blind study using ANCOVA.

## Results

### Subject Characteristics

Of the 84 subjects enrolled in the double-blind study, 6 subjects were ineligible to enroll in the open-label study due to early terminations or serious AEs. Four subjects decided not to participate due to moving away from the study site, decision with the caregiver not to enroll due to lethargy, intensity of protocol, and lost to follow-up. Although one subject who withdrew from the double-blind study early due to pre-existing breast cancer was initially excluded, once medically cleared, she was permitted to enroll, making a total of 75 subjects who enrolled in the open-label study (Table 1). Three of the 75 subjects were excluded from analysis because they terminated at or prior to the week 12 visit. Forty-two subjects completed the 80-week extension and washout period (Figure 1).

**Table 1:** Baseline characteristics (n = 75)

Characteristic	
Age (mean ± SD, range)	50.9 ± 11.5, 29.2-77.4

CAGn (mean ± SD, range)	44.5 ± 3.4, 39-54

Women, n (%)	49 (65%)

Caucasian, n (%)	71 (95%)

Affected Parent, n (%)	
Mother	33 (44%)
Father	33 (44%)
Unspecified	9 (12%)

Years of Illness duration (mean ± SD, range)	8.5 ± 4.5, 1.9-25.9

History of Depression, n (%)	42 (56%)

**Figure 2:**
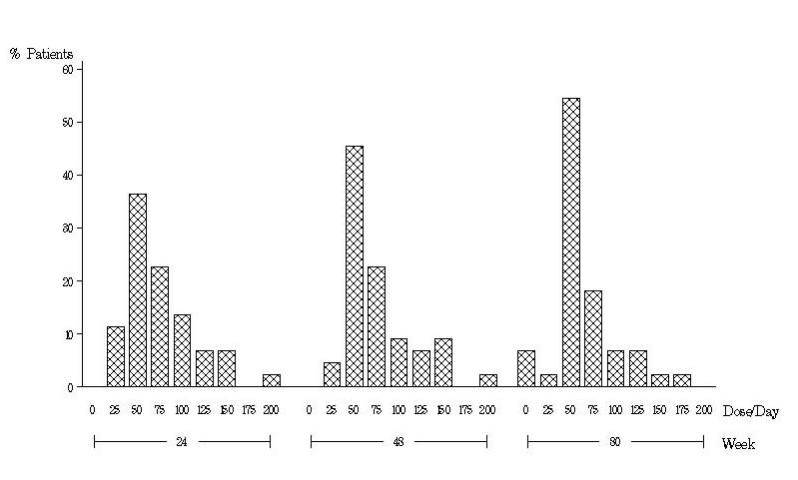
**Dosage distribution by percent of subjects at weeks 24, 48 and 80 for the 44 subjects who continued to take tetrabenazine through week 80**.

Excluding subjects who had stopped medication, the mean daily dosage at week 24 (n = 66) was 74.2 mg (± 40.9, range 12.5 mg-200 mg), week 48 (n = 54) was 71.5 (± 41.5, range 12.5 mg-200 mg) and at week 80 (n = 41) was 63.4 mg (± 34.2, range 12.5 mg-175 mg). For the 44 subjects with complete dosage data at week 80 (including three subjects with zero dose), 24 (55%) of participants were taking either 37.5 mg or 50 mg per day (Figure 2). Regarding adjustment of dosage for subjects who completed 80 weeks, 7 (16%) increased their dosage between weeks 24 and 48, 31 (70%) stayed on the same dose, and 6 (14%) decreased. Between weeks 48 and 80, 6 (14%) of these subjects increased their dosage, 26 (59%) stayed on the same dose, and 12 (27%) decreased (including 3 with week 80 dose = 0 who washed out early).

### Tolerability

Twenty subjects terminated from the study. There was one death due to metastatic breast cancer. Three participants withdrew due to AEs attributed by the investigators to TBZ, including vocal tics, depression, and delusions. One subject terminated because of an elevated bilirubin level but also reported akathisia, while another subject experienced a significant increase in liver enzymes. One subject was started on exclusionary medications, and two subjects were non-compliant with study medications. One subject was institutionalized, one moved out of the state, and two were lost to follow-up. The remaining seven terminations were due to withdrawal of patient consent or by caregiver or physician request. An additional ten subjects decided not to continue in ensuing extension phases. Subjects who completed the study were more likely to be female (p < 0.01), have higher CAG repeat length (45 vs. 43.5, p < 0.05), and have a lower total functional assessment score at baseline (18.0 vs. 20.5, p < 0.02). There were no differences in race, affected parent, history of depression, chorea score, TFC, total motor score, age, years of education or duration of illness.

There were 12 serious AEs including two falls, two cancer diagnoses, a single suicide attempt, pneumonia, hip replacement (elective) with post-op agitation, agitation, anxiety, akathisia and one abnormal CA 27-29 titer in a participant who later died due to metastatic breast cancer. During the study, 56 subjects reported 170 AEs. Seventeen subjects reported depressed mood as an AE during this study with a prior history of depression in 15 of those subjects. All AEs with an incidence of 5% or more are listed in Table 2. There was no association between any adverse event and concomitant medications.

**Table 2:** Treatment emergent AEs reported in >5% of subjects (3 or more), excluding mild and not related to treatment

Adverse Event	# of Subjects
Sedation/Somnolence	18

Depressed Mood	17

Anxiety	13

Insomnia	10

Akathisia	9

Fatigue	7

Agitation	5

Fall	4

Dysphagia	3

Dystonia	3

When mild or unrelated events were excluded, 39 subjects reported at least one AE during titration while 20 subjects reported at least one AE during maintenance (p < 0.001). Insomnia, somnolence and diarrhea emerged during titration and subsequently resolved during maintenance. The number of subjects with somnolence decreased from 36 to 11 (p < 0.0001), insomnia from 14 to 2 (p < 0.003) and diarrhea from 5 to 1 (p < 0.05) when comparing titration to maintenance phases.

### Safety Measures

Between baseline and week 80, the mean parkinsonism score increased 2.1 (SD 4.3) UHDRS units (p = 0.002) and the mean UPDRS dysarthria score increased 0.4 (SD 0.8) UPDRS units (p < 0.002). There were no significant changes in the HAM scale scores, UPDRS dysphagia score, or BAS. Marked or severe akathisia (4 or 5 on the BAS scale) was experienced by only one participant, who terminated due to elevated bilirubin, rather than akathisia. Three participants (4%) had mild or moderate akathisia at baseline. Ten other participants (13.3%) developed mild or moderate akathisia during the course of the study.

There were 3 participants with isolated elevation of AST, greater than 3 times the upper limit of normal. Two occurrences were at baseline and one at week 24. All abnormal liver tests returned to normal by week 80 or at the end of study participation except in one participant with >2 times upper limit of normal AST in isolation. No participant experienced clinical liver dysfunction, but one participant was terminated early due to elevated AST and ALT at baseline and one subject terminated due to elevated bilirubin without clear etiology.

### Efficacy

**Figure 3:**
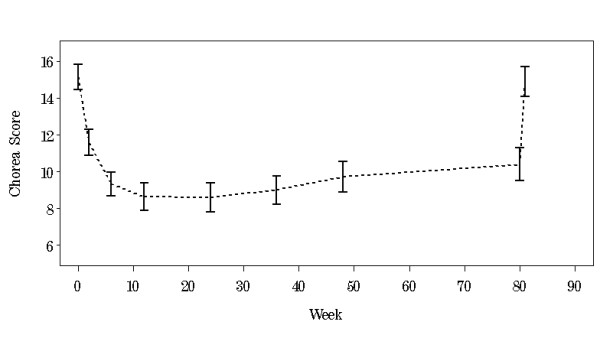
Mean total maximal chorea score (± SE) by week

When TMC at week 80 was compared to baseline, there was a reduction in mean TMC score by 4.6 (SD 5.5) UHDRS units (p < 0.001). At week 81 after washout, chorea re-emerged with a mean TMC score increase of 5.3 (SD 3.2) UHDRS units (p < 0.001 compared to week 80) (Figure 3). For the 41 participants with complete data, there was no difference in TMC at week 81 (14.9 ± 5.1) when compared to baseline (15.1 ± 4.3).

At 48 weeks, chorea reduction in the 45 participants who completed 80 weeks was not significantly different compared to the 11 subjects who completed week 48 but did not complete the study. After adjusting for study site and baseline chorea score, there was no association with the effect of TBZ on chorea related to age, gender, trinucleotide repeat length, gender of affected parent, or baseline clinical global impression of severity. Baseline chorea score itself was a predictor for greater reduction in chorea (p = 0.013).

### Secondary Efficacy Outcomes

At week 80, there was a significant change in the CGI score of 0.3 (SD 0.7, p = 0.0054). Of the 45 completers, there was improvement in 16 (36%) and worsening of 4 (9%) with the remainder unchanged compared to baseline.

In the 45 participants who completed 80 weeks, there was no significant change in total motor score, but there were significant declines in cognitive measures, including verbal fluency, symbol digit and Stroop (reading, color and interference) and functional measures as measured by the functional checklist, independence score and TFC (Table 3).

**Table 3:** Baseline scores and changes from baseline in efficacy outcome measures at weeks 24, 48 and 80 [Mean (SD)].

Measure	Baseline	Week 24	Week 48	Week 80
	**N = 75**	**N = 73**	**N = 57**	**N = 45**

UHDRS Motor Score	47.9 (15.9)	-7.4 (9.8) *	-5.6 (12.1) *	-0.2 (11.0)

Total Chorea	14.9 (3.7)	-5.8 (5.0) *	-5.6 (5.5) *	-4.6 (5.5) *

Gait	1.3 (0.6)	-0.1 (0.6)	0.0 (0.7)	0.2 (0.7)

Parkinsonism	14.5 (5.7)	-0.5 (2.7)	0.9 (4.1)	2.1 (4.3) *

Barnes Akathisia	0.2 (0.6)	0.1 (1.0)	0.1 (0.9)	0.0 (0.7)

Hamilton	4.1 (3.7)	-0.0 (3.8)	0.7 (3.4)	0.4 (3.8)

Functional Checklist	18.1 (4.7)	-0.3 (2.5)	-1.1 (2.3) *	-2.6 (3.3) *

Independence	75.7 (11.5)	-2.4 (7.2) *	-4.4 (6.0) *	-7.0 (8.1) *

Behavioral Score (freq*severity)	6.7 (9.7)	3.4 (14.4) *	2.9 (9.3) *	3.5 (14.4)

Weight (kg)	72.4 (18.8)	-1.2 (4.8) *	-1.4 (7.4)	-1.7 (6.5)

	**N = 47**	**N = 45**	**N = 38**	**N = 30**

Total Functional Capacity	7.6 (2.4)	-0.5 (1.5) *	-1.1 (1.4) *	-2.0 (2.3) *

Verbal	17.2 (10.3)	-2.0 (5.6) *	-2.0 (5.8) *	-3.6 (7.9) *

Symbol Digit	19.3 (11.2)	-0.3 (5.5)	-1.9 (4.7) *	-4.8 (10.3) *

Stroop Color	42.6 (17.3)	-4.4 (9.5) *	-8.9 (11.4) *	-11.0 (14.8) *

Stroop Words	49.8 (20.6)	-1.2 (9.3)	-7.9 (15.4) *	-9.0 (17.4) *

Stroop Interference	23.2 (11.4)	-2.7 (7.1) *	-4.5 (7.0) *	-5.0 (10.4) *

* p < 0.05 (t-test)

### Placebo vs. TBZ

When the groups initially randomized to placebo and TBZ were compared, there were no differences in numbers of subjects with AEs, change in TMC score, change in chorea after withdrawal, best dosage, or any of the secondary measures. There was a reduction in the total number of AEs (p < 0.002) and somnolence (p < 0.01) in maintenance for those subjects initially assigned to TBZ.

## Discussion

The double-blind, randomized controlled trial preceding this study demonstrated that TBZ reduced chorea associated with HD for up to 12 weeks with the main AEs including drowsiness and insomnia [9]. In this open label extension study, TBZ continued to effectively suppress chorea for up to 80 weeks in HD. TBZ was generally well tolerated, but treatment emergent AEs included sleep disturbance, depression, anxiety, and akathisia. Akathisia in particular may be difficult to identify due to confusion with chorea combined with the lack of insight associated with HD. During initiation and titration of therapy, patients may be more prone to sedation, insomnia and gastrointestinal problems but these tend to improve over time. Although clinicians should screen for and treat depression in all patients with HD, those with pre-existing depression may be at higher risk for developing depression while taking TBZ [23,24].

In this study, there were no completed suicides, but one attempt listed as a serious AE and an additional subject who expressed suicidal ideation. Depression was not assessed by formal psychiatric criteria, but depressed mood was screened for using the Hamilton Depression Scale and item 25 of the UHDRS. During the double-blind phase of this study, there was a completed suicide in a subject randomized to the TBZ group, despite scoring within the normal range on the Hamilton Depression Scale two weeks prior to the event [9]. Clinicians should be cautious about the heightened risk of suicide in HD regardless of depression indexes or use of monoamine depleters, as overall completed suicide rates are estimated at 7.3% and up to 25% of patients may attempt suicide at some point in the illness [25,26]. In patients already diagnosed with HD, there may be an increased risk of suicide particularly when patients enter stage 2 of the disease, a time when independence diminishes [27].

At the conclusion of this study, the majority of participants were taking either 50 or 75 mg of TBZ. Notably, even after subjects had been on TBZ for over one year, dosage adjustments continued. In two other open-label, long term studies of TBZ used for a variety of hyperkinetic conditions including HD, the mean doses after more than two years were 60.4 and 106.2 mg, respectively [6,7].

Discontinuation of TBZ after 80 weeks of treatment appears to be safe and is associated with the return of chorea, without significant worsening compared to baseline. Participants with higher baseline chorea scores experienced a greater reduction in chorea. When TBZ was discontinued, no subject developed signs consistent with neuroleptic withdrawal such as nausea, excessive sweating, tachycardia or akathisia. We did not identify AEs related to the withdrawal of TBZ, but other studies have specifically examined subjects when TBZ is stopped [9,28]. One subject withdrew due to the development of "vocal tics". This AE has not been previously reported and in fact TBZ has been found to be an effective treatment of tics [29]. It is possible that the "vocal tic" described was not a TBZ-related AE, but the emergence of tourettism as a symptom of HD [30].

Participants did not demonstrate a faster than expected rate of motor or functional decline due to HD. Although parkinsonism and dysphagia were increased in subjects at week 80, this finding was likely a result of the natural progression of disease. Had the parkinsonism been due to a pharmacological impact of tetrabenazine, we would have anticipated that the signs would have emerged more quickly and found on exams throughout the study. TBZ does not appear to have an effect on cognition or function after two years of therapy. Participants experienced an overall improvement on TBZ as demonstrated by the CGI at week 80, but there was no correlation to support the association of global improvement with either motor or chorea change. The UHDRS measures of cognition and function while taking TBZ declined at a rate consistent with the natural history of HD. The TFC scale declined 1.6 ± 0.4 points over almost two years in those initially assigned to TBZ, consistent with previously published measures of TFC [31,32]. Although TBZ is unlikely to accelerate any aspect of decline in HD, it also does not appear to have any clinical long-term neuroprotective role, despite some evidence of potential neuroprotective effects demonstrated in animal models of HD [33].

The main limitation of this open-label study was the loss of subjects at each extension phase. The reasons for the attrition were related to administrative issues and a decision not to continue rather than any specific AEs. The majority of patients elected to complete 80 weeks on therapy, and in fact, once the study concluded, some subjects refused to stop taking TBZ and found other sources of the study medication prior to its availability on the US market. The findings support the clinical impression that chorea suppression in this large cohort of patients can be valuable to patients and families. The unblinded nature of this study limits the degree to which we can make conclusions about effectiveness, but the reduction in chorea was consistent with that seen in the double-blind trial. Further investigation is needed to determine the effectiveness and adverse event profile, particularly regarding cognition, when TBZ is combined with other commonly used medications.

## Conclusions

Chorea, the most striking physical manifestation of HD, may be disabling and stigmatizing. Frequent or large amplitude involuntary movements can cause embarrassment or frustration and physical harm due to direct injury or falls as well as impact quality of life. The impact of suppressing chorea on weight, gait, behavior and functioning deserves further study, as do the indications for suppressing chorea in the ambulatory high functioning patient and the more advanced patient in whom chorea may be injurious. In lieu of discovering therapy that modifies the global course of the disease, identifying targeted symptomatic treatments for HD, such as those for chorea, is a way to palliate some of the more disabling and stigmatizing aspects of the disorder. This study confirms efficacy in long term use and may guide clinicians in dosing and treatment emergent, long-term adverse effects.

## Competing interests

This study was funded by a grant from Prestwick Pharmaceuticals, Inc. (subsequently acquired by Biovail Pharmaceuticals) to the University of Rochester and in turn through subcontracts to the participating research sites. The Huntington Study Group (HSG) is a non-profit consortium of HD investigators http://www.huntington-study-group.org/. None of the HSG investigators or staff had equity interests with Prestwick Pharmaceuticals, Inc. Dr. Fahn received consulting fees of less than $10,000 from Prestwick Pharmaceuticals. After the study was completed, Dr. Frank received consulting fees of less than $10,000 from Ovation Pharmaceuticals. Dr. Marshall presented data at meetings for which he received travel reimbursement from Prestwick Pharmaceuticals, Inc. Dr. Stamler and Ms. Wilson were employees of Prestwick Pharmaceuticals, Inc. The HSG Coordination and Biostatistics Centers at the University of Rochester independently compiled and analyzed the data for this study.

## Pre-publication history

The pre-publication history for this paper can be accessed here:

http://www.biomedcentral.com/1471-2377/9/62/prepub
