# Correction:Tetrabenazine as anti-chorea therapy in Huntington Disease: an open-label continuation study

**DOI:** 10.1186/1471-2377-11-18

**Published:** 2011-02-04

**Authors:** Samuel Frank

**Affiliations:** 1 72 East Concord St., C329, Boston, MA 02118, USA

## Correction

After publication of this work [1], we became aware of a typographical error. The term dysphagia inadvertently replaced the term dysarthria in two instances. Dysarthria was statistically significantly worsened at week 80 compared to baseline, whereas there was only a statistical trend (p = 0.10) for dysphagia at week 80 compared to baseline. Dysphagia continues to be an issue in patients with Huntington Disease and was reported as an adverse event in this study. Therefore, clinicians who prescribe tetrabenazine should still monitor their patients for worsening swallowing, but the worsening was not statistically different in those who completed this trial. No further studies of tetrabenazine in Huntington Disease have been completed since the publication of this study.

Dysarthria should be replaced for dysphagia in two places and the manuscript should read as follows:

Abstract, page 1:

Results: Parkinsonism and **dysarthria **scores were significantly increased at week 80 compared to baseline.

Discussion, page 8:

Although parkinsonism and **dysarthria **were increased in subjects at week 80, this finding was likely a result of the natural progression of disease.

The other uses of the terms dysarthria and dysphagia in the manuscript are correct. We regret the error and any inconvenince it may have caused.

## Pre-publication history

The pre-publication history for this paper can be accessed here:

http://www.biomedcentral.com/1471-2377/11/18/prepub
